# Editorial: Organ-sparing surgery for genitourinary cancers

**DOI:** 10.3389/fonc.2024.1443878

**Published:** 2024-07-18

**Authors:** Gongwei Long, Xingyuan Xiao, Haoran Liu, Yucong Zhang, Chunguang Yang

**Affiliations:** ^1^ Department of Urology, National Cancer Center/National Clinical Research Center for Cancer/Cancer Hospital & Shenzhen Hospital, Chinese Academy of Medical Sciences and Peking Union Medical College, Shenzhen, China; ^2^ Department of Urology, Zhongnan Hospital, Wuhan University, Wuhan, China; ^3^ Department of Chemistry and Bio-X, Stanford University, Stanford, CA, United States; ^4^ Department of Chemistry, The University of Hong Kong, Hong Kong, Hong Kong SAR, China; ^5^ Department of Geriatrics, Tongji Hospital, Tongji Medical College, Huazhong University of Science and Technology, Wuhan, China; ^6^ Department of Urology, Tongji Hospital, Tongji Medical College, Huazhong University of Science and Technology, Wuhan, China

**Keywords:** organ-sparing surgery, genitourinary cancers, radical cystectomy, bladder cancer, renal tumor

Genitourinary cancers, including renal cancer, bladder cancer, prostate cancer, and other malignancies within the urinary and reproductive system, are common diseases with an increasing incidence worldwide (1). Traditionally, for localized lesions, radical surgeries would be recommended for patients for curative purposes. Although radical therapy is still the primary option for cancer treatment, organ-sparing surgeries were increasingly accepted by surgeons and patients, due to the significantly improved prognosis in recent decades and the better quality of life after organ-sparing treatments.

For example, radical cystectomy is the standard surgical treatment for muscle-invasive bladder cancer, but as the most morbid and complex urologic surgery, radical cystectomy could bring postoperative complications to over half of patients, and about 1-4% of patients might die within 90 days after surgery, let alone the severely impaired quality of life (2). Recently, bladder-preserving therapies have been applied to selected patients. With effective systemic treatments, radical cystectomy might be waived for these patients who achieved complete response while the long-term outcomes were not compromised compared to radical cystectomy (3–5). On the other hand, surgical modifications were also explored. Maximal elimination of bladder tumors by transurethral En bloc resection could be more informative and curative, and might be the new gold standard of transurethral surgery in the new era (6–8). For tumors unfit for transurethral resection, radical cystectomy is not inevitable, as partial cystectomy could be offered for selected patients, and the prognosis is also acceptable (9).

For renal tumors, nephron-sparing surgery has been the recommended treatment for patients with localized tumors due to the equivalent oncological outcomes compared to radical nephrectomy (10, 11). While for more advanced tumors, evidence suggests that partial nephrectomy is still optional for selected patients (12, 13).

Besides, organ-sparing surgeries have also been attempted in other genitourinary tumors, including prostate cancer (14), penis cancer (15), and rare malignancies such as urachal carcinoma (16). For now, organ-sparing surgeries provide additional options for part of patients, and might bring a better balance between cancer control and quality of life. Therefore, this topic is proposed to share the highlight works in organ-sparing surgery, as well as relevant neoadjuvant and adjuvant therapy, for the treatment of genitourinary cancers.

As mentioned above, bladder-preserving therapy has been widely used in muscle-invasive bladder cancer. Al-Qudimat et al. conducted an updated meta-analysis to compare the oncological outcomes of trimodal therapy with radical cystectomy in muscle-invasive bladder cancer, and emphasized the important role of standard radical cystectomy in the management of muscle-invasive bladder cancer. Ghali et al. also focused on the trimodal therapy of bladder cancer, and they found that over a third of patients ≥65 years old received curative-intent radiotherapy alone without concurrent chemotherapy. These two studies alert us that trimodal therapy should be applied according to a more standard protocol with caution. Yin et al. reported a case of bladder squamous cell carcinoma treated with partial cystectomy. This patient remains free of disease recurrence and metastasis 10 years after partial cystectomy, which might provide some reference for the individualized treatment of bladder cancer. Radical cystectomy could sometimes be offered to high-risk non-muscle-invasive bladder cancer patients who experience repeating recurrence. Photodynamic therapy could be an alternative option before radical cystectomy, but the lack of solid research fundaments limited its wide use. Li et al. conducted a systematic review to comprehensively summarize the application of photodynamic therapy in non-muscle-invasive bladder cancer. This study confirmed the safety and efficacy of photodynamic therapy, and warrants further high-quality research to expand the use of it as well.

As for renal tumors, Yan et al. presented their laparoscopic aspirator bracket which could help the aspiration and exposure of the operative field simultaneously during the laparoscopic partial nephrectomy. Li et al. shared their experience of super selective transarterial embolization of renal tumors. This technique allows a more precise blockage of tumor blood supply and zero-ischemia robotic-assisted laparoscopic partial nephrectomy without renal arterial clamping.

In this Research Topic, several studies reported their updated research outcomes, mainly focused on the organ-sparing strategies in bladder cancer and renal cancer. Alternative treatments to avoid radical surgery, switching radical surgery to less damaging organ-sparing surgery, and surgical technique modification were attempted by these studies to preserve the organ. Also, like other organ-sparing strategies, these efforts were preliminary, and future work is warranted to ascertain the beneficiary and standard treatment protocols.

As a promising strategy, organ-sparing surgeries would be more commonly accepted in the future. We confidently look forward to further high-quality studies to solve puzzles in this field and provide more feasible options for patients who suffer from genitourinary cancers.

## Author contributions

GL: Writing – original draft. XX: Writing – review & editing. HL: Writing – review & editing. YZ: Writing – review & editing. CY: Writing – review & editing.

## References

[1] Siegel RL Giaquinto AN Jemal A. Cancer statistics, 2024. CA Cancer J Clin. (2024) 74:12–49. doi: 10.3322/caac.21820 38230766

[2] Djaladat H Katebian B Bazargani ST Miranda G Cai J Schuckman AK. 90-Day complication rate in patients undergoing radical cystectomy with enhanced recovery protocol: a prospective cohort study. World J Urol. (2017) 35:907–11. doi: 10.1007/s00345-016-1950-z 27734131

[3] Giacalone NJ Shipley WU Clayman RH Niemierko A Drumm M Heney NM. Long-term outcomes after bladder-preserving tri-modality therapy for patients with muscle-invasive bladder cancer: an updated analysis of the massachusetts general hospital experience. Eur Urol. (2017) 71:952–60. doi: 10.1016/j.eururo.2016.12.020 28081860

[4] Mazza P Moran GW Li G Robins DJ Matulay JT Herr HW. Conservative management following complete clinical response to neoadjuvant chemotherapy of muscle invasive bladder cancer: contemporary outcomes of a multi-institutional cohort study. J Urol. (2018) 200:1005–13. doi: 10.1016/j.juro.2018.05.078 PMC7543664 29787740

[5] Shi H Zhang W Bi X Wang D Xiao Z Guan Y. Neoadjuvant chemotherapy-guided bladder-sparing treatment for muscle-invasive bladder cancer: results of a pilot phase II study. Cancer Res Treat. (2021) 53:1156–65. doi: 10.4143/crt.2020.1356 PMC8524034 33592141

[6] Teoh JY-C MacLennan S Chan VW-S Miki J Lee H-Y Chiong E. An international collaborative consensus statement on en bloc resection of bladder tumour incorporating two systematic reviews, a two-round delphi survey, and a consensus meeting. Eur Urol. (2020) 78:546–69. doi: 10.1016/j.eururo.2020.04.059 32389447

[7] Long G Zhang Y Sun G Ouyang W Liu Z Li H. Safety and efficacy of thulium laser resection of bladder tumors versus transurethral resection of bladder tumors: a systematic review and meta-analysis. Lasers Med Sci. (2021) 36:1807–16. doi: 10.1007/s10103-021-03272-7 33604772

[8] Liu Z Long G Zhang Y Sun G Ouyang W Wang S. Thulium laser resection of bladder tumors vs. Conventional transurethral resection of bladder tumors for intermediate and high risk non-muscle-invasive bladder cancer followed by intravesical BCG immunotherapy. Front Surg. (2021) 8:759487. doi: 10.3389/fsurg.2021.759487 34820417 PMC8606824

[9] Long G Hu Z Liu Z Ye Z Wang S Wang D. Partial and radical cystectomy provides equivalent oncologic outcomes in bladder cancer when combined with adequate lymph node dissection: A population-based study. Urol Oncol. (2023) 41:327.e1–8. doi: 10.1016/j.urolonc.2023.02.004 36966065

[10] Zhang Y Long G Shang H Ding B Sun G Ouyang W. Comparison of the oncological, perioperative and functional outcomes of partial nephrectomy versus radical nephrectomy for clinical T1b renal cell carcinoma: A systematic review and meta-analysis of retrospective studies. Asian J Urol. (2021) 8:117–25. doi: 10.1016/j.ajur.2019.11.004 PMC7859367 33569278

[11] Mir MC Derweesh I Porpiglia F Zargar H Mottrie A Autorino R. Partial nephrectomy versus radical nephrectomy for clinical T1b and T2 renal tumors: A systematic review and meta-analysis of comparative studies. Eur Urol. (2017) 71:606–17. doi: 10.1016/j.eururo.2016.08.060 27614693

[12] Tian J Zeng X Wan J Gan J Ke C Guan W. Partial and radical nephrectomy provides equivalent oncologic outcomes in pT3a renal cell carcinoma: A population-based study. Front Oncol. (2021) 11:819098. doi: 10.3389/fonc.2021.819098 35155208 PMC8826755

[13] Tian J Zeng X Zhu J Guan W Hu Z Yang C. Cytoreductive partial and radical nephrectomies provide equivalent oncologic outcomes in T1-2M1 renal cell carcinoma. Transl Cancer Res. (2023) 12:301–9. doi: 10.21037/tcr-22-1389 PMC10007872 36915574

[14] Villers A Puech P Flamand V Haber G-P Desai MM Crouzet S. Partial prostatectomy for anterior cancer: short-term oncologic and functional outcomes. Eur Urol. (2017) 72:333–42. doi: 10.1016/j.eururo.2016.08.057 PMC9084627 27613061

[15] Scarberry K Angermeier KW Montague D Campbell S Wood HM. Outcomes for organ-preserving surgery for penile cancer. Sex Med. (2015) 3:62–6. doi: 10.1002/sm2.56 PMC4498822 26185670

[16] Ke C Xu L Wang M Wang Y Zhou Q Chen J. Treatment options and prognostic risk factors for urachal carcinoma: A multicetnter retrospective study. Urol Oncol. (2023) 41:50.e1–9. doi: 10.1016/j.urolonc.2022.09.017 36283930

